# The Optimal Permeation of Cyclic Boronates to Cross the Outer Membrane via the Porin Pathway

**DOI:** 10.3390/antibiotics11070840

**Published:** 2022-06-23

**Authors:** Gian Marco Tuveri, Matteo Ceccarelli, Alessandro Pira, Igor V. Bodrenko

**Affiliations:** 1 Molecular Bionics, Institute for Bioengineering of Catalonia, Carrer de Baldiri Reixac, 10, 12, 08028 Barcelona, Spain; gtuveri@ibecbarcelona.eu; 2 Dipartimento di Fisica, University of Cagliari, Cittadella Universitaria, Monserrato, 09042-IT Cagliari, Italy; matteo.ceccarelli@dsf.unica.it; 3 Centro Nazionale di Ricerca/Istituto Officina dei Materiali (CNR/IOM), Sezione di Cagliari, c/o Dipartimento di Fisica, Cittadella Universitaria, Monserrato, 09042-IT Cagliari, Italy; 4 Dipartimento di Scienze Chimiche e Geologiche, University of Cagliari, Cittadella Universitaria, Monserrato, 09042-IT Cagliari, Italy; alessandro.pira@dsf.unica.it; 5 Department of Life Sciences and Chemistry, Jacobs University Bremen, Campus Ring 1, 28759-DE Bremen, Germany

**Keywords:** cyclic boronates, beta-lactamase inhibitors, porins, permeation, diffusion current, molecular dynamics simulations, metadynamics

## Abstract

We investigated the diffusion of three cyclic boronates formulated as beta-lactamase inhibitors through the porin OmpF to evaluate their potential to cross OM via the porin pathway. The three nonbeta-lactam molecules diffuse through the porin eyelet region with the same mechanism observed for beta-lactam molecules and diazobicyclooctan derivatives, with the electric dipole moment aligned with the transversal electric field. In particular, the BOH group can interact with both the basic ladder and the acidic loop L3, which is characteristic of the size-constricted region of this class of porins. On one hand, we confirm that the transport of small molecules through enterobacter porins has a common general mechanism; on the other, the class of cyclic boronate molecules does not seem to have particular difficulties in diffusing through enterobacter porins, thus representing a good scaffold for new anti-infectives targeting Gram-negative bacteria research.

## 1. Introduction

Modern medicine requires the constant development of anti-infectives to counteract the innate resistance of bacteria [1]. In the second half of the last century, medicinal chemistry fueled the golden age of antibiotics through the chemical modification of existing scaffolds. A large number of antibiotics available was not accompanied by the discovery of new scaffolds, thus favouring the spread of resistance [2]. Today, innovative strategies are necessary to discover new scaffolds for combating infections and blocking the spread of resistant bacteria [3]. This problem is exacerbated by its spread at the global level and an industrial pipeline that is virtually empty, especially for Gram-negative bacteria [4,5].

Because of the difficulties of developing new scaffolds, an alternative strategy points to the development of adjuvants, which can improve the efficacy of already available antibiotics and, thus, revitalize their use [6]. This is the case with beta-lactamase inhibitors (BLI) [7,8,9], which reinforce beta-lactam antibiotics, namely penicillins, cephalosporins, and carbapenems. These inhibitors are able to bind beta-lactamase enzymes more strongly than antibiotics, avoiding their chemical disruption and allowing them to reach the target [10]. In particular, in recent years, we have remarked the discovery of new and very effective non-beta-lactam scaffolds, such as diazobicyclooctan derivatives [11]. Boron-containing compounds have increased in the last few years their appeal in biomedical research, particularly in the drug design sector [12]. Recently, researchers discovered boron-containing, non-beta-lactam cyclic boronate (CB) molecules that appear to have good beta-lactamase inhibition effects [13,14,15].

The problem of the discovery of new antibiotics is particularly urgent for Gram-negative species [16], protected by an additional outer membrane (OM). Acting as a real physical barrier [17], OM limits the penetration of compounds and their arrival on internal targets [18,19]. Thus, its action is general and provides a non-specific resistance affecting a wide range of antibiotics: the so-called multidrug resistance [20]. In several years of investigations to rationalize compound permeation to cross the OM, strategies, for example, based on post-analysis of molecular properties failed [21], arriving at the conclusion that antibiotics targeting Gram-negative bacteria do not look similar to other drugs, described quite well by the Lipinski rule of 5 [22]. One limitation to the discovery of new scaffolds combating Gram-negative species is then the lack of rules for identifying molecules with a good permeation, which is the main reason for the difficulty in measuring and quantifying the permeation of molecules through the OM [23,24]. The use of accumulation data in the cell [25,26], indirectly related to permeation, and molecular modeling [27] combined with single-channel experiments has provided a few molecular rules for explaining the permeation of compounds through the OM; however, they are not yet assessed or robust enough to implement in a high-throughput search.

Recently, these molecular rules were combined to develop a scoring function for predicting the permeability of compounds through porins, supported by a well-defined physical interaction model [28]. Although the scoring function is based on general molecular properties [29], namely the size, the charge, and the electric dipole moment, the molecules selected for its fitting were all beta-lactam antibiotics, which are considered molecules with good permeation [28]. Investigations on diazobicyclooctan derivatives [30] confirmed, by molecular modeling, that the main contribution to an efficient permeation comes from the size, charge state, and electric dipole moment of molecules, irrespective of the chemical scaffold [28]. In addition, for the development of ETX0462 the molecular dipole moment was monitored to improve rationally its permeation through the OM [31].

The scoring function was further tested on a large set of non-anti-infectives molecules for which accumulation data were available. The high correlation obtained between accumulation and prediction, 74% on 137 molecules [32], pointed out two key questions: (i) the generality of the scoring function; since accumulation is highly correlated to permeation, (ii) porins still represent the main path of entry in Gram-negative bacteria [33].

Another approach for predicting the permeability of compounds would be the use of artificial intelligence algorithms, which are becoming very popular in drug discovery [34]. However, these methods require a large set of data and this conflicts in some way with the difficulty in measuring permeation [23]. A very recent contribution on permeability in Pseudomonas aeruginosa employed data from 83 molecules to apply a machine learning algorithm that generated eventually a deterministic classification model [35]. However, these methods do not provide new knowledge in terms of the mechanism of permeation, apart from the classification of molecules in clusters. Thus, the approach of the scoring function seems to have two advantages: (i) Numerically speaking, it can be applied with a small data set of molecules, being in the functional form of the interaction known; (ii) it is based on well-defined molecule-pore interactions, coming from robust knowledge of the mechanism of permeation. Thus, we can apply it to any molecule and pore via the use of specific physical/chemical parameters. The existence of new CB beta-lactamase inhibitors represents a more tight test for investigating the permeation of small molecules through porins and, thus, validating the scoring function, since these molecules need to overcome OM to reach the target enzymes.

**Figure 1:**
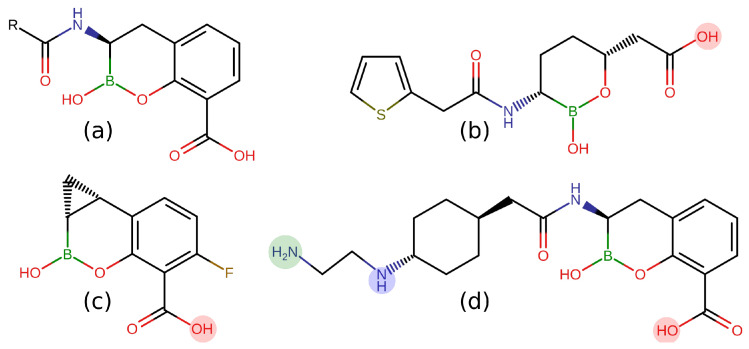
Lewis structures of the cyclic boronates in 2 dimensions: (**a**). precursor [13]; (**b**). Vaborbactam [13], carboxyl group deprotonated (charge −1) at pH 7.4; (**c**). QPX7728 [14], carboxyl group deprotonated (charge −1) at pH 7.4; (**d**). taniborbactam [15], carboxyl group deprotonated, secondary amine group protonated (total charge 0, zwitterionic) at pH 7.4 with probability 51% (tanibor0); in addition, the primary amine group can be also protonated with probability 34% at pH 7.4 (green circle, tanibor1, and total charge +1).

With the aim to understand how CB scaffolds can penetrate through porins, we selected three recent BLIs (Vaborbactam, Taniborbactam, and QPX7728, see Figure 1) [13,14,15], and we investigated their diffusion through model porin OmpF. By applying state-of-the-art accelerated molecular dynamics simulations, we showed how the boronate ring interacts with the constriction region of the OmpF eyelet, thus conferring efficient permeation to this scaffold.

## 2. Results and Discussion

*Simulations in bulk water.* The OmpF pore, the first bacterial porins solved at high resolution, represents a model system for describing enterobacteriaceae bacterial porins [28]. These beta-barrel proteins are characterized by an hourglass shape, created by the internal folding of the loop L3 at mid-height, and show the so-called separation of charge. The latter is due to the presence of basic residues on one side (the basic ladder) facing the acidic loop L3 on the other, thus creating a large transversal electric field [36] to which molecules align when diffusing through [37]. For these reasons, two key molecular parameters for diffusion are the size of molecules and their dipole moment [30].

**Figure 2:**
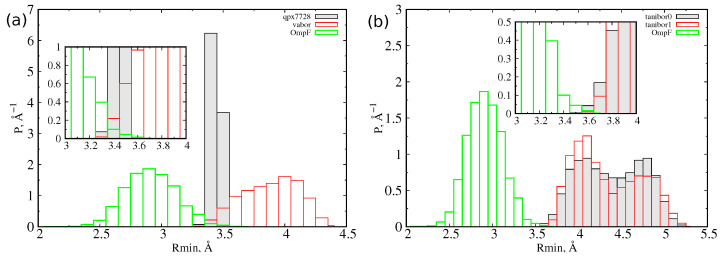
(**a**) Probability distribution function of the $R_{min}$ for QPX7728, vaborbactam, and OmpF. (**b**) Probability distribution of the $R_{min}$ for the two tautomers of taniborbactam and OmpF. In the inset, the overlap region of distributions is highlighted.

**Figure 3:**
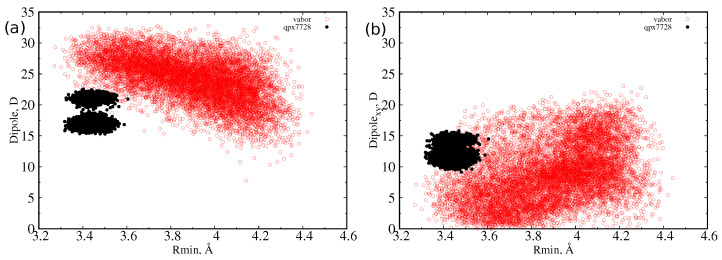
Scatter plot of dipole moment and minimum radius for vaborbactam (red) and QPX7728 (black) as sampled during the MD simulations in water. (**a**) Total dipole moment versus minimum radius; (**b**) transversal dipole moment versus minimum radius.

At first, we let the three CB inhibitors (Figure 1) explore their conformational space in a box of water to obtain statistical descriptions of the size and dipole moment. For quantifying the size of a molecule, we used the minimal projection area (MPA) and the associated minimum radius $R_{min}$, as described in detail in Section 3. MPA represents the minimal cross sectional area of a molecule projected on a plane, different for any possible conformation assumed by the molecule itself. From molecular dynamics simulations of the inhibitors in water, we calculated, for each sampled conformation, the MPA and the corresponding minimum radius, and we reported the probability distribution function of the latter in Figure 2. The characteristic values of these distributions are collected in Table 1. QPX7728 is the molecule with the smallest average radius (<R> = 3.4 Å). However, we see in the inset that also the large (on average) vaborbactam (<R> = 3.9 Å) can attain a radius as small as that of QPX7728, although with a lower probability, overlapping with the OmpF size distribution. In addition, the larger scaffold of vaborbactam can bring a larger dipole moment, as reported in Figure 3.

**Figure 4:**
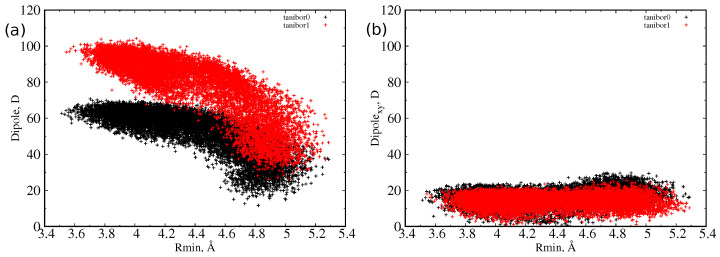
Scatter plot of dipole moment and minimum radius for taniborbactam0 (red) and taniborbactam1 (black) as sampled during MD simulations in water. (**a**) Total dipole moment versus minimum radius; (**b**) transversal dipole moment versus minimum radius.

For taniborbactam, we compared the two most probable tautomers at neutral pH, the zwitterionic form (tanibor0), and the positively charged form (tanibor1). Taniborbactam is (on average) larger than the other two CBs and shows two peaks in the distribution, corresponding to an extended shape (R${}_{mode}$ = 4.0 Å) and a compact shape (R${}_{mode}$ = 4.7 Å). The addition of a positive charge has a negligible effect on the size, with the charged form showing a higher probability of the extended shape. From the inset of Figure 2, we see that the overlap with OmpF’s size is weaker than it is for the other two BLIs. As for vaborbactam, the larger dimension of taniborbactam confers to this scaffold a very large strength of the dipole moment, as remarked by the scatter plot of Figure 4, which varies from 60 Debye to almost 100 Debye or an increment of 50% upon the addition of the positive charge.

However, as already observed [30], the dipole reaches its largest value at the lowest value of the minimum radius, or when the molecule is extended, an indication of a longitudinal dipole. This is confirmed by the analysis of the dipole moment components with respect to the MPA plane. We defined the transversal dipole moment (Dipole${}_{xy}$) as the component of the dipole projected on the plane perpendicular to the main axis (that is, the xy or MPA plane). In Figure 3 and Figure 4, it is shown that, as the radius decreases, the dipole does not grow in the MPA plane. This implies, as already explained, that the only increasing component is the one along the molecules’ major axis. This effect is greater for the largest molecule, taniborbactam. It is worth to note how QPX7728 has its dipole moment mainly in the transversal direction, being the total dipole almost equal to the transversal one; see Figure 3. From Table 1, we see that the average Dipole${}_{xy}$ for QPX7728 is larger than in vaborbactam, and among the two tautomers, the zwitterionic one has a larger transversal dipole than the charged one, although the total dipole moment performs the opposite.

**Figure 5:**
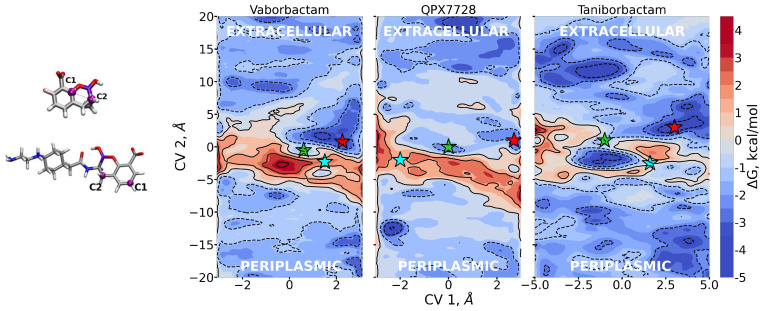
Two-dimensional free energies as obtained by metadynamics simulations of the three inhibitors diffusing through OmpF. High energy values are defined by warmer colors. CV1 measures the orientation of the molecules with respect to the *z* diffusion axis; CV2 its position along the *z* diffusion axis. We identify with stars the key regions of the free-energy surfaces. The corresponding conformers are exposed in Figure 6, Figure 7 and Figure 8.

*Simulations within the OmpF.* Since the diffusion of molecules through pores occurs on time scales of several microseconds [38], in order to sample their diffusion with molecular simulations, we need to employ accelerated techniques [39]. Here, we performed metadynamics simulations to observe and sample the diffusion of the BLIs through OmpF. As performed in the past [30], we biased two coordinates: (i) CV1 is related to the orientation of the boronate ring (Figure 5, left panel) with respect to the *z* diffusion axis; (ii) CV2 represents the position of BLIs boronate ring along the *z* diffusion axis. Thanks to these CVs, we can force the orientation of molecules to change at the entrance of the pore, thus sampling more efficiently the relative orientation also in the constricted region, where there is no space for reorienting. For vaborbactam, we used the same atoms as in QPX7728 to define CV1, while for taniborbactam, we took different atoms.

From metadynamics, we can reconstruct the free energy surface with respect to the biased CVs, thus allowing the quantification of free energy barriers and affinity sites encountered by the three BLIs during diffusion. The free energies are reported in Figure 5. As expected, FESs show free energy barriers around CV2 = 0, indicating the overlap between the center of mass of porin and CBs, since the pore is constricted roughly in the middle in correspondence to the folding of loop L3.

**Figure 6:**
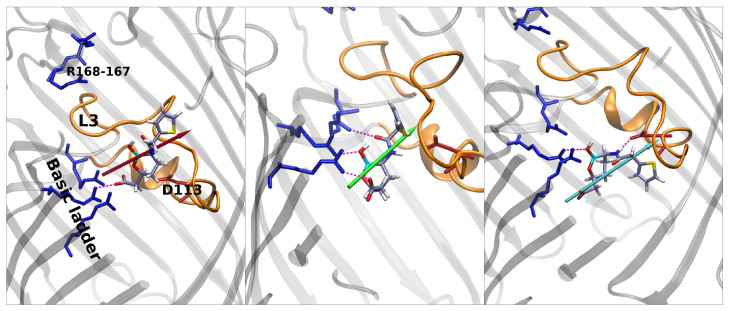
From **left** to **right**: main conformers noticed in porin for vaborbactam. The color scheme of dipoles follows the FES in Figure 5. Vaborbactam enters the constriction region firstly with the carboxyl group that interacts strongly with the basic ladder residues. The molecule slides inside OmpF, aligning the dipole to the CR electric field. The full alignment allows the BLI to increase its MPA inside the CR.

**Figure 7:**
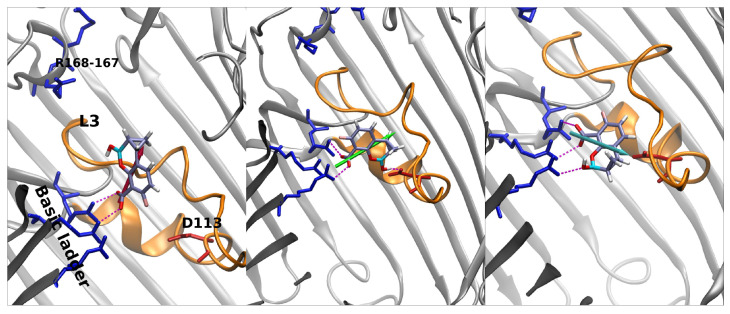
From **left** to **right**: main conformers extracted when QPX7728 diffuses through the pore’s CR. The color scheme of dipoles follows the FES in Figure 5. QPX approaches the basic ladder with the carboxyl group and then rotates along its major axis maintaining this bond and building a new one between the BOH group and Asp-113.The rotation continues and brings the BOH group to interact with the ladder. This last configuration allows the molecule to cross the CR.

**Figure 8:**
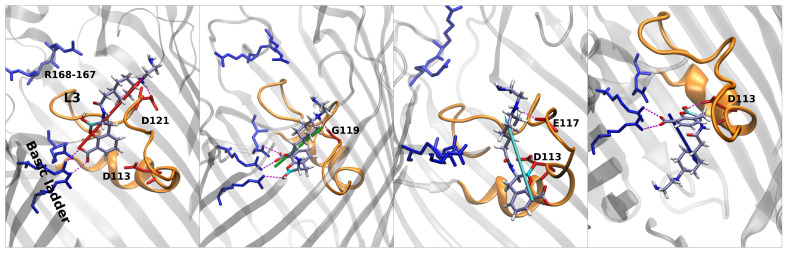
From **left** to **right**: main conformers noticed in the porin for taniborbactam. The color scheme of dipoles follows the FES in Figure 5. Taniborbactam interacts strongly with the CR through its charged groups and the BOH group. The large size is compensated by a great dipole that is aligned to the CR’s electric field. Once at the lower part of the CR, the BLI’s dipole is not aligned to the field for reducing the hindrance.

In Figure 5, the stars represent special regions of FESs identified by the combination of the two collective variables, corresponding either to affinity sites or saddle points; the corresponding conformers are shown in Figure 6, Figure 7 and Figure 8, and they follow the same color scheme, with the arrows of a given color indicating the corresponding dipole moment at that site. A thorough analysis of these conformers permits the elucidation of the molecular mechanism of diffusion through the OmpF pore.

Vaborbactam shows an energy peak in the exact center of the FES, which is the excluded region. CV1 = 0 implies that the z-component of the vector shown in Figure 5 is zero; i.e., the antibiotic is oriented orthogonal to the diffusion axis. This could be hard for vaborbactam to achieve, because of the great steric hindrance this configuration would create. The red star in the basin in vaborbactam’s FES (Figure 5) corresponds to the first conformation in Figure 6. Here, vaborbactam enters in contact with the upper part of the constriction region (CR) through the interaction of the deprotonated carboxylic group with the basic ladder. This stable interaction is visited several times during the calculations, and the dipole moment (red arrow) is aligned to the transversal electric field of the pore. From here, vaborbactam slides toward CR, keeping the dipole moment aligned to the electric field. The system assumes a stable configuration in the middle of FES, identified by the green star in Figure 5; the corresponding green dipole in Figure 6 indicates the boronate ring is almost orthogonal to the diffusion axis. Now, group BOH also interacts with the basic ladder, and this interaction is important to keep the molecule near CR. For this conformation, an important step is to cross the CR with the thiophene ring, while the BOH group keeps the interaction with the basic ladder. Finally, the interaction between vaborbactam’s amide-NH and Asp-113 allows the molecule to overcome CR (cyan star in Figure 5, cyan dipole in Figure 6). The increase in steric hindrance is compensated by the alignment of the dipole moment with the electric field. In the mechanism, the thiophene ring is involved in the crossing mechanism, although it does not interact directly with CR.

QPX7728 approaches the CR as vaborbactam (positive CV1), but the passage is more likely to happen for negative values of CV1. QPX7728’s FES in Figure 5 shows two stable configurations (red and green star) and a saddle point (cyan star), which is the most likely trajectory to cross the barrier. These conformations are shown in Figure 7. The first contact of QPX7728 with the CR is again an interaction of the deprotonated carboxyl group with Arg-132 and Arg-82 (red star). The molecule then rotates, keeping the latter interaction as a pivot. The second stable conformation (green star) is defined by a double interaction of QPX7728’s carboxyl with Arg-42 and Arg-82 and the BOH group with Asp-113. To reduce the steric hindrance and permit the passage, QPX has to rotate again. By keeping the first interaction, the oxygen of the BOH group, such as the carboxyl group, can interact with the basic ladder. This rotation with respect to the molecular major axis and the basic ladder as a pivot allows the small molecule to overcome CR. It is important to note that, during the entire path described, the dipole is aligned to the transversal constriction region’s electric field. Furthermore, the BOH group plays an important role in the mechanism, allowing the molecule to stay in contact with CR.

Regarding taniborbactam, we simulated only the zwitterionic tautomer, tanibor0 (see Figure 1d). We saw that this molecule possesses a size larger than the other two CBs (Figure 2); however, it was accompanied by a larger dipole moment, (Figure 4). Furthermore, this molecule has secondary amine groups that can effectively interact with the acidic L3 loop. Indeed, the low values of energy obtained in the barrier are also the result of this peculiarity. In Figure 5, we observe a first stable region in the extracellular side of the pore, above the barrier (red star). Figure 8 shows that also taniborbactam prefers to enter CR with the deprotonated carboxylic group. This group interacts with the basic ladder while the secondary amine group interacts with the upper part of L3, specifically Asp-121. From this configuration, the system moves to the central basin in Figure 5 (green star) with a rotation of the boronate ring with the carboxylic group as a pivot, just as observed for QPX7728. This conformation has a bigger MPA, but this is fully compensated by a large number of interactions. In particular, the oxygen of the BOH group creates a hydrogen bond with the basic ladder (Arg-42), while the secondary amine group performs the same with the backbone of the L3 residue Gly-119. Finally, the system reaches the final conformation, depicted with a cyan star. Here again, the interaction of BOH with Asp-113 takes place. This conformer shows that, to cross the barrier, the molecule has to momentary align the dipole against the electric field. The direct passage of the molecule from the red to the cyan state is also possible: The molecule only has to slide, as observed with vaborbactam. Taniborbactam is peculiar, and the presence of the large energy minimum in the middle of the FES is also peculiar (Figure 5, which is created by the antibiotic trying to cross the CR in the opposite direction, from trans to cis. This is visible from the last conformer in Figure 8. The interactions with the basic ladder and Asp-113 (in particular with the BOH group) are strong and create a deep affinity site for taniborbactam. However, the conformer’s dipole is not perfectly aligned to the electric field, and BLI’s orientation has a large steric hindrance that prevents taniborbactam from crossing CR in the wrong direction.

**Figure 9:**
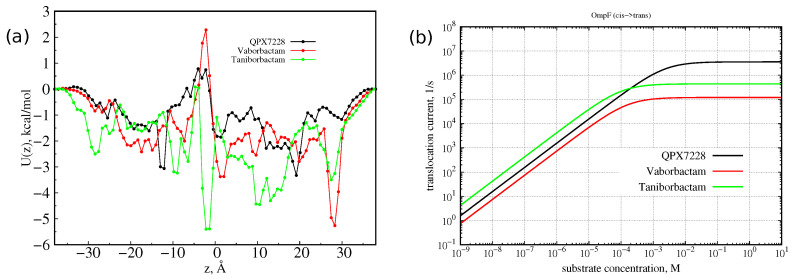
(**a**) One-dimensional FESs for the three molecules with respect to the CV2 and their position along the *z* diffusion axis. (**b**) Predicted diffusive current of molecules over the one-dimensional FESs versus the external gradient concentration (the molecules substrate is added to cis side).

*Predictions of currents.* From the two-dimensional FESs of Figure 5, we calculated the free energies along the *z* diffusion axis only, reported in Figure 9, by statistical averaging over variable CV1. The one-dimensional FESs show that the molecule with the highest barrier, with respect to the bulk, is vaborbactam, followed by QPX7728 and Taniborbactam. In CR and before entering it, taniborbactam shows higher affinity with the pore: Being zwitterionic, it has a larger dipole moment that aligns to the transversal electric field of OmpF [27,36], better compensating the steric free energy barrier. The other two anionic molecules have similar free energies, with more affinity for vaborbactam.

From the one-dimensional FES, it is also possible to predict the current of diffusing molecules versus the external gradient concentration. At a low substrate concentration, one can neglect the interaction between the substrate molecules. Then, diffusion is described by the linear 1D Smoluchowski equation, and the diffusion current is proportional to the substrate concentration gradient, as given by a Kramers-type integral formula [40]. As performed previously [30], the high substrate concentration condition was treated with a two-state Markov model [40]; we used value of 1 nm${}^{2}$/ns for the effective diffusion constant. This model assumes that, at the maximum, one particle at a time may occupy the channel. It can describe both the linearity with the concentration and the saturated behavior of the particle translocation regime. The current of molecules as a function of gradient concentration is reported in Figure 9. Because of the small barrier shown by the three molecules, the current at 1 µM concentration is a few thousand molecules per second, larger than the value measured for avibactam [41], having a dipole similar to QPX7728 and larger size. The molecule with the highest current is taniborbactam, followed by QPX7728 and vaborbactam, following the trend of the increasing central barrier. The high affinity of taniborbactam with OmpF is also visible in Figure 9b. Its current saturates already at 100 μM, at 1 mM vaborbactam, and then at almost 10 mM does QPX7228. To note, these concentration values are larger than the typical one expected around bacteria.

In the linear regime (at low concentration limit), the translocation diffusive current is proportional to the concentration gradient (Fick’s law) and is the same from cis to trans and from trans to cis given the same concentration gradient. At high concentration gradients, the saturated (maximum) translocation current in Figure 9b is equal to the $I^{\max}\left(cis\to trans\right)=k_{\mathrm{off}}^{\mathrm{trans}}$ rate of the Markov state model [40]. By using the electrophysiology of a single OmpF channel reconstituted in an artificial lipid bilayer membrane [27], one can, in principle, determine the average dwell time, $\tau_{b}$, when the ionic current through the pore is blocked (or reduced) due to the presence of the molecule inside. As reported in detail in [40], we have the following:

(1) $$\begin{array}{ccc} \tau_{b} & = & \frac{1}{k_{\mathrm{off}}^{\mathrm{trans}}+k_{\mathrm{off}}^{\mathrm{cis}}}=\frac{1}{I^{\max}\left(cis\to trans\right)+I^{\max}\left(trans\to cis\right)}, \end{array}$$

and we can estimate the dwell time for antibiotics as being well below 1 μs. These fast events were not observed experimentally for vaborbactam (M. Winterhalter private communication) using standard single-channel single-molecule electrophysiology techniques. The predicted dwell times in the sub-microsecond range requires the use of specific techniques to measure permeability, either with the use of the reversal potential method for charged molecules [41], or measuring current at diverse concentration gradients for neutral molecules [38]. Thus, as we showed here, accelerated molecular dynamics simulations represent a useful tool to investigate the diffusive transport of molecules in the fast regime and discuss their mechanism.

*Structure and permeation.* The results obtained here on the three CB confirmed that the mechanism of permeation is found for other scaffolds and permits establishing some principle of permeation, also with respect to other findings. (i) As the hour-glass shape of enterobacteriaceae porins suggests, a good permeate molecule should have a cylindrical shape, with a small minimal projection area, even with a long extension. This condition can be also satisfied by a fluctuating shape and not necessarily by a rigid one. The limit of 600 Dalton indicated, years ago [42], that it is in fact referred to compact and rigid molecules, as demonstrated by a recent investigation on molecules with mass exceeding the 600 Dalton [43]. (ii) Because of the small size of the pore constriction, any molecule will encounter a steric barrier to diffuse through it [44]. Thus, energetically, the diffusion is unfavored. However, the existence of an electric field inside porins would favor those molecules with a dipole moment that can align to the electric field [37]. In order to satisfy at best the alignment condition between a transversal electric field and a cylindrical scaffold, the dipole moment should be transversal to the axis of the cylinder. The case of taniborbactam analyzed here is instructive since we showed that the two tautomers, although differing in the total dipole moment by a large amount (40 Debye), have exactly the same transversal dipole moment, as shown in Figure 4. The presence of a boronic acid group together with a carboxylic group in the scaffold allows having a good transversal dipole moment that is necessary to compensate the steric barrier. Any positive charge added to the lateral chain has no effect on the transversal dipole moment and only on the longitudinal dipole moment and the total charge. Furthermore, we saw that during diffusion, the boronic rings are the first part of the molecules that interact with CR, see Figure 8, while the lateral chain interacts with the upper part of the loop L3. (iii) It is interesting to point out that two negatively charged molecules, as vaborbactam and QPX7728, can permeate well OmpF porin. This seems to contrast with the suggestion that the addition of a positive charge improves permeation and thus accumulation [25]. This is only apparently in contrast, since the addition of a positive charge enhances the electric dipole moment of a molecule, and the better permeation/accumulation would be explained by a better compensation due to alignment with the internal electric field of porins. We might speculate that the condition to have a positive charge is a sufficient condition for having good permeation; this is not necessary as it is just a method to improve the dipole moment. On the contrary, the presence of a large transversal dipole moment is a necessary condition for compensating the steric term and, thus, improve permeation, as we observed for taniborbactam, which is the molecule with the largest size investigated here. (iv) The condition to have a large dipole moment is in conflict with having a small MPA [32]. Indeed, the dipole moment is defined as the separation of charged groups. Small scaffolds by constraints would posses small dipole moment; thus, the condition of optimal permeation is a subtle balance between having not too large MPA and not too small transversal dipole moment. The use of the scoring function can in fact quantify both contributions, allowing an optimization of the two quantities [28], with a high prediction power [32].

## 3. Methods

*Boronic acid parametrization.* Parametrization of the cyclic boronates (CBs) was conducted using the GAFF force field [45] in antechamber [46], after obtaining an initial structure of each CB with MarvinSketch [47]. Gaussian09 Revision A.02 [48] was used to perform the ground-state geometry optimization of the main tautomer for each CB in implicit solvent (Polarizable Continuum Model), by applying the Density Functional Theory (DFT) with a hybrid exchange-correlation functional B3LYP, and 6-31G** Gaussian basis-set. We doubly verified the CBs’ charge configuration with MarvinSketch and OpenBabel [49], taking the tautomer with the highest probability at pH 7.4. Only for taniborbactam did we decide to test the two most likely charge configurations, since they have similar probability at pH 7.4. Once we fixed the total charge of each CB, atomic partial charges were calculated on the optimized conformations with Gaussian using HF/6-31G* functions in vacuum. The charges were then generated through the two-step restrained electrostatic potential (RESP) method [50], as implemented in the Antechamber package. In order to avoid errors with antechamber’s atomic type recognition, the boron atom has been treated as an sp2 carbon, which shows the same number of planar bonds as B in CB. In the end, we created a new empiric force field for boron in a cyclic structure, using as equilibrium parameters (bond lengths, bond, and dihedrals angles) the optimized geometry values and GAFF sp2 carbon as force constants.

We used MarvinSketch to calculate the Minimal Projection Area MPA. For this physical quantity, we associated a minimum radius defined as $R_{min}={\left(MPA/\pi \right)}^{\frac{1}{2}}$, assuming MPA has a circular shape. Moreover, for evalutating the size of the pore, we calculated the surface available to a probe of radius 1.4 Å, and then we took the associated minimum radius defined as $R_{min}={\left(SURFACE/\pi \right)}^{\frac{1}{2}}$. Moreover, in this case, the circular approximation applies.

*Molecular Dynamics Simulations.* Each CB was initially solvated with TIP3P water [51] in a cubic box with edges of 30 Å, adding NaCl ions for charge compensation up to the physiological concentration of 0.15 M. Then, we proceed with a minimization–equilibration cycle with molecular dynamics simulations at all-atom level. After a minimization of 2 ps, the solvated CBs were gradually heated to equilibrium from 10 K to 300 K, with an intermediate step at 50 K, in a NVT ensemble; each step was 100 ps long. We then ran the first set of simulations in the NPT ensemble at a temperature of 300 K for 1 µs. We used these initial data to calculate CBs’ size and dipole moment.

Regarding pore simulation, we started from the X-ray structure of OmpF as initial coordinates (PDB entry 2OMF). All polar residues are in the estimated charge configuration at physiological pH 7.4, with the only exception of residue Glu-296 that was protonated as proposed by Varma et al. [52]. The OmpF trimer was embedded in a POPC (1-palmitoyl-2-oleoyl-sn-glycero-3-phos-phocholine) bilayer of 259 units and solvated with a 200 mM KCl solution, in addition to an excess of 33 potassium cations to compensate the negative charge of OmpF; the system was minimized and equilibrated as previously described [28]. The protein was parametrized with amberff14SB [53] for proteins, GAFFlipid [54] for POPC, TIP3P [51] for explicit water, the General Amber FF [45] for standard atoms, and Boronic Acid FF for the boron (non-standard) atoms, as described above. The initial box has a dimension of 108×108×87 Å${}^{3}$, with OmpF occupying the centre of the box and its axis along the direction *z*. We used ACEMD [55] with the Langevin thermostat to keep the temperature constant, with damping 0.1 ps${}^{-1}$. Long-range interactions were treated with the particle mesh Ewald (PME), with cutoff 9 Å and switch at 7.5 Å. The dynamics of CBs with OmpF were accelerated with the Well-Tempered Metadynamics as implemented in the PLUMED plugin [56]. As collective variables, we selected two variables. The first is the orientation of the boronate ring along the *z* axis: Because of the rigidity of the structure, this is the perfect indicator for the orientation of the entire molecule. The vector is the same for vaborbactam and QPX7728, which is the distance between the boron next-neighbor carbon (C${}_{2}$) and the opposite carbon in the ring (C${}_{1}$). For taniborbactam, we have chosen a different distance, considering that the boron ring is bonded to an aromatic ring that is important in interactions. Thus, as CV1, we considered the *z* component of the vector ${\vec{C}}_{2}$-${\vec{C}}_{1}$ shown in the left panel of Figure 5. CV2 is the position of the molecular center of mass of each CB along the diffusion axis *z*. We prepared OmpF trimer + CB system positioning the molecule at the entrance of the first monomer (residues 1–340), at a distance of 25 Å along *z* between the two centers of mass and deleting the overlapping water molecules. CBs are biased to move along the *z* axis inside a cylinder of radius 17 Å that shares its axis with the first OmpF monomer. This forces CBs to proceed through the first monomer of the porin, exploring the conformational space without losing the generality of the phenomenon. All metadynamics simulations were performed in the NVT ensemble at 300 K; all bonds with hydrogens were treated as rigid, and masses of hydrogen were rescaled to allow a timestep of 4 fs [57].

Because of the independent evolution of each replica in multiple walker metadynamics simulations, we used 4 to 12 replicas depending on the availability of computational resources. The total simulation times are reported in Table 2.

## 4. Conclusions

We demonstrated that cyclic boronate scaffolds can penetrate well through bacterial porins from the Enterobacteriaceae family. The mechanism that we highlighted for diffusing through OM is the same one found for other molecules, although with a different scaffold. The three molecules approaches the CR with the deprotonated carboxylic group before; then, the boronate ring interacts with CR. The molecular dipole moment when aligned to the transversal electric field provides a non-negligible compensation for the steric barrier created by the restricted size of porins, as remarked for taniborbactam. This molecule, the largest among the three, shows a very low effective barrier for diffusion. The large compensation comes from a large dipole moment, made possible by the large size of the scaffold. This explains the apparent paradox that molecules with small sizes do not have an intrinsic better permeability through size-constricted pores, as those represented by OmpF.

In general, all CBs show favorable interactions inside CR. The main reasons is the presence of a transverse dipole, capable of aligning to the inner electric field of OmpF. Our data show that CB’s ability of adjusting the dipole to the electric field is correlated to the structure of the boronic ring. The ring possesses, in all CBs, a carboxyl group (deprotonated at physiological pH) and a hydroxyl group bonded the boron atom: the BOH group. While the carboxyl group can interact only with basic residues, the BOH group is highly reactive and can share hydrogen bonds with both the acidic and basic amino acids. This is a strong motivation to search for containing-boron scaffolds that, when designed to bind directly to the beta-lactam target, might work as antibiotics with good permeation; thus, they are effective against Gram-negative bacteria.

The current of molecules driven by the gradient concentration is linear at physiological concentrations, larger for the zwitterionic taniborbactam versus the anionic vaborbactam and QPX7728. On the other hand, saturation occurs before for the zwitterionic taniborbactam. The tested CB molecules diffuse in fast regime; thus, accelerated molecular simulations are an invaluable tool to characterize their permeation and mechanism. Eventually, our results corroborate the idea found for other scaffolds that, in order to enhance permeation through enterobacteriaceae porins, we need to increase the transversal dipole moment by chemical modifications. The addition of a positive charge is not a necessary condition for good permeation; instead, it works in the direction that is able to improve the transversal dipole moment. Thus, also negative molecules, as shown here, can have good permeation.

## Figures and Tables

**Table 1:** The table reports, for each of the four solvated BLIs, the values of *R*${}_{min}$ and dipole moment characterizing the distributions. For the two taniborbactam’s tautomers, the distributions are bimodal and we reported both mode values.

BLI	*R*${}_{min}$ Mean, Å	*R*${}_{min}$ Mode, Å	Dipole Mean, D	Dipole${}_{xy}$ Mean, D
QPX7728	3.4	3.4	17.7	12.0
vaborbactam	3.9	4.0	24.2	8.9
tanibor0	4.4	4.0–4.7	52.4	16.4
tanibor1	4.3	4.0–4.7	79.5	14.0

**Table 2:** Simulation time in μs for the analyzed BLIs in OmpF porin. The total metadynamics simulation times are specified with the concerning number of Replicas. All the steps are carried with Well-Tempered Metadynamics.

	QPX7728		Vaborbactam		Taniborbactam	
	Time	Replicas	Time	Replicas	Time	Replicas
	700 ns	4	700 ns	1	1000 ns	1
	1800 ns	8	2000 ns	12	1300 ns	12
	/		/		6200 ns	8
Total	17.2 µs		24.7 µs		66.2 µs	

## Abbreviations

The following abbreviations are used in this manuscript:

CR	Constriction Region;
FES	Free energy surface;
BLI	Beta-lactamase Inhibitors;
OM	Outer Membrane;
CB	Cyclic Boronate;
MPA	Minimal Projection Area.
